# Large-Scale and Localized Laser Crystallization of Optically Thick Amorphous Silicon Films by Near-IR Femtosecond Pulses

**DOI:** 10.3390/ma13225296

**Published:** 2020-11-23

**Authors:** Kirill Bronnikov, Alexander Dostovalov, Artem Cherepakhin, Eugeny Mitsai, Alexander Nepomniaschiy, Sergei A. Kulinich, Alexey Zhizhchenko, Aleksandr Kuchmizhak

**Affiliations:** 1 Institute of Automation and Electrometry of the SB RAS, 1 Acad. Koptyug Ave., 630090 Novosibirsk, Russia; bronnikovkirill@gmail.com (K.B.); alexdost@gmail.com (A.D.); 2 Novosibirsk State University, 2 Pirogova St., 630090 Novosibirsk, Russia; 3 Institute of Automation and Control Processes FEB RAS, 5 Radio St., 690041 Vladivostok, Russia; cherepakhinab@yandex.ru (A.C.); eugenemitsai@gmail.com (E.M.); santila001@mail.ru (A.N.); g89leksig@mail.ru (A.Z.); 4 Far Eastern Federal University, 690041 Vladivostok, Russia; skulinich@tokai-u.jp; 5 Research Institute of Science and Technology, Tokai University, Hiratsuka, Kanagawa 259-1292, Japan

**Keywords:** amorphous silicon, polycrystalline silicon, thin films, laser-induced annealing, femtosecond laser pulses, Raman spectroscopy

## Abstract

Amorphous silicon (α-Si) film present an inexpensive and promising material for optoelectronic and nanophotonic applications. Its basic optical and optoelectronic properties are known to be improved via phase transition from amorphous to polycrystalline phase. Infrared femtosecond laser radiation can be considered to be a promising nondestructive and facile way to drive uniform in-depth and lateral crystallization of α-Si films that are typically opaque in UV-visible spectral range. However, so far only a few studies reported on use of near-IR radiation for laser-induced crystallization of α-Si providing less information regarding optical properties of the resultant polycrystalline Si films demonstrating rather high surface roughness. The present work demonstrates efficient and gentle single-pass crystallization of α-Si films induced by their direct irradiation with near-IR femtosecond laser pulses coming at sub-MHz repetition rate. Comprehensive analysis of morphology and composition of laser-annealed films by atomic-force microscopy, optical, micro-Raman and energy-dispersive X-ray spectroscopy, as well as numerical modeling of optical spectra, confirmed efficient crystallization of α-Si and high-quality of the obtained films. Moreover, we highlight localized laser-induced crystallization of α-Si as a promising way for optical information encryption, anti-counterfeiting and fabrication of micro-optical elements.

## 1. Introduction

Due to the advantages of polycrystalline silicon (poly-Si) over its amorphous counterpart (α-Si), which include orders of magnitude higher carrier mobility and better stability, thin films of poly-Si have become the basis for solar cells, thin-film transistors, high definition displays, vertically integrated memory devices, etc. [1,2,3,4,5]. Large scale poly-Si is traditionally obtained in a chemical reaction of hydrogen reduction of trichlorosilane SiHCl${}_{3}$ in rod-type reactors (so-called “Siemens process”) or by pyrolytic decomposition of monosilane SiH${}_{4}$ in rod-type and boiling-layer-type reactors [6]. However, these methods include high-temperature exposure (700–1000 ${}^{\circ }$C), thus restricting the choice of substrates to refractory materials and, therefore, cannot be used to produce low-temperature polycrystalline silicon (LTPS) which is currently the most promising material for polymer-based flexible displays requiring high-speed operation and high resolution [4]. Crystallization of amorphous silicon is an actively studied approach for obtaining poly-Si thin films since thin films of α-Si can be produced by cheap low-temperature chemical or physical vapor deposition over large-scale substrates including glasses and flexible polymers, that allows to extend functionality and reduced fabrication cost of resulting optoelectronic devices. To improve optical properties and inherently low carrier mobility of α-Si films, multiple efforts were undertaken to perform conversion of amorphous Si material to its polycrystalline phase. More specifically, crystallization of α-Si was realized using thermal annealing [7], solid-phase crystallization [8], metal-induced crystallization and modifications of this method for lateral crystallization [9,10]. All these methods are usually accompanied by a melting-cooling-solidification cycle.

In recent decades, laser annealing was recognized as a flexible technique to locally crystallize α-Si with high control [11]. In particular, the use of excimer laser with ultraviolet (UV) radiation was previously justified to efficiently drive transition of amorphous Si to its polycrystalline phase allowing to produce high-quality films [12,13,14,15]. However, ultra-low penetration depth of UV light to silicon limits the thickness of α-Si film, which can be processed with such an approach. Visible laser radiation (in particular, blue and green lasers operating in CW and pulsed mode [1,16,17,18,19,20]) was shown to increase the maximal processing thickness to 150–200 nm; however, this is still not enough to cover all applications. Noteworthy, penetration depth of near-IR radiation into Si, which has good transparency in this spectral range (comparing to UV and visible light), is high enough to drive crystallization inside rather thick α-Si films that are required for realistic applications and devices. As an example, CW diode lasers with λ ≈ 800 nm with line-shaped focal point are widely used to obtain poly-Si absorber layers in solar cells through liquid-phase crystallization [21,22,23,24,25]. In these reports, typically α-Si films deposited on glass substrate are as thick as around 10 μm. During the process, the substrate is usually heated up to 600–700 ${}^{\circ }$C to avoid cracking and reduce the laser fluence required to cause melting, which is around 100–200 J/cm${}^{2}$ [26]. So far, numerous studies were reported which consider laser crystallized silicon thin-film solar cells reaching power conversion efficiency as high as 13–14% [22,24,25]. In addition, UV pulsed-laser treatment was applied to crystallize Si emitter layers [27] or create contact points of the solar cell and increase doping of Si absorber near contacts [28]. However, CW or down to nanosecond pulsed regimes exploited in these applications require relatively high fluence levels to initiate the melting and do not offer precise spatial material processing [29,30].

Femtosecond radiation opens up new possibilities in laser-induced crystallization exploiting nonlinear pulses energy absorption that results in high spatial localization of material modification and low fluence threshold for crystallization in comparison with longer pulses due to minimization of heat diffusion [31,32]. It was previously found that the fluence threshold depends on the laser wavelength, being lower when using the second harmonics (λ = 400 nm), which was explained by a shallower melting depth in the case of shorter wavelengths [33]. It was also shown that, in comparison with other experimental parameters (pulse duration, polarization, number of pulses, etc.), the substrate temperature significantly affects the crystallization process reducing the threshold down from 65 mJ/cm${}^{2}$ to 49 mJ/cm${}^{2}$ when increasing the temperature from 25 to 200 ${}^{\circ }$C [34]. Various dependencies of the poly-Si characteristics on the laser processing parameters were reported recently: the size of the crystallites [35], crystallization area homogeneity [36], photoelectric properties [37], and surface morphology [38]. So far, only a few studies reported on use of near-IR radiation for laser-induced crystallization of α-Si films providing information regarding optical properties of the poly-Si films [39,40,41]. Among these, Hong et al thoroughly investigated optical properties of Si films crystallized with fs-pulsed laser, aiming at enhanced solar cell performance due to laser-assisted formation of surface structures at fluencies of several hundreds of mJ/cm${}^{2}$ [41]. However, increased surface roughness may be undesirable in some applications, which is why a sparing low-energy α-Si crystallization regime is needed.

Here, we used near-IR femtosecond laser pulses to directly drive phase transition of glass-supported 365-nm thick α-Si, which resulted in formation of high-quality poly-Si film with low surface roughness. Elliptically shaped laser beam with an aspect ratio of 10 was used to improve the uniformity of laser-crystallized areas. Composition and crystallinity of obtained films were verified with Raman and energy-dispersive X-ray spectroscopy, while comparative atomic-force microscopic (AFM) analysis of the produced poly-Si and as-deposited α-Si samples revealed a very small increase of surface roughness from 0.5 to 1.3 nm upon laser texturing. Optical spectroscopic studies supported by numerical modeling confirmed improved optical characteristics of laser-annealed films in the visible spectral range associated with reduction of the absorption coefficient associated with polycrystalline phase. Direct IR femtosecond laser processing was demonstrated to uniformly anneal α-Si over large-scale surface area or enable local phase transition, opening pathway for various applications ranging from solar cells and optoelectronics to microoptics and information storage.

## 2. Materials and Methods

α-Si films with a thickness of 365 ± 5 nm were deposited onto a borosilicate glass plate by magnetron pulsed DC sputtering. The chamber was evacuated to a pressure of 4 × 10${}^{-3}$ Pa prior to deposition. Argon was used as discharge gas for sputtering at a pressure of 0.13 Pa. During sputtering, the power was fixed at 320 W. Substrate-to-target distance was set to 27 mm and deposition time was 129 s. Films were used for laser annealing without any pre-treatment. Direct laser-induced crystallization of such films was performed using femtosecond (pulse duration τ = 230 fs) IR (wavelength λ = 1026 nm) laser pulses generated by a regeneratively amplified Yb:KGW laser system (Pharos, Light Conversion Ltd., Vilnius, Lithuania) at a maximal pulse repetition rate *f* of 200 KHz.

**Figure 1:**
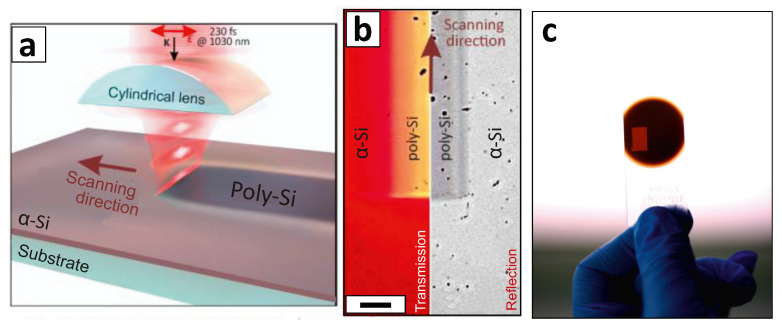
(**a**) Scheme of laser-induced annealing of α-Si films. (**b**) Reflection and transmission optical microscope images of produced poly-Si stripe surrounded by pristine α-Si film. Scale bar indicates 40 μm. Multiple black spots on reflection image represent surface debris. (**c**) Optical image of uniform 1 cm${}^{2}$ poly-Si area recorded in α-Si film.

The Gaussian-shaped beam generated by the laser system was passing through a cylindrical concave lens with the focal distance of –1 m and then was focused by a convex lens with the focal distance of 50 mm (Figure 1a). The resulting intensity distribution in the focal plane presented elliptically shaped beam with a size of 160 μm along long axis and the axes ratio of 1:10. Based on our previous studies [42], pulse energy *E*${}_{p}$ (peak laser fluence *F*${}_{l}$) and scanning speed *V* were chosen to be 1.5 μJ (150 mJ/cm${}^{2}$) and 1 mm/s, respectively. The samples were arranged onto a 2D motorized platform (Aerotech Gmbh., Nurnberg, Germany) permitting patterning of rather large surface areas by merging with a certain overlap line scans produced with the elliptical-shaped laser beam. Localized laser-annealing was performed by focusing the IR laser radiation with a dry microscope lens with a numerical aperture of 0.8 that yielded in 1/e-diameter of the laser spot in the focal plane ≈1 μm. Same fluence and scanning speed were used to record microscale poly-Si areas.

Laser-annealed areas of the α-Si film were carefully characterized by scanning electron microscopy (SEM, Ultra 55+, Carl Zeiss, Oberkochen, Germany) equipped with an energy-dispersive X-ray detector (EDX; X-max, Oxford Instruments, Abingdon, UK), as well as atomic-force microscopy (AFM, Nano-DST, Pacific Nanotechnology, Santa Clara, CA, USA). Transmission and reflection spectra in the ultraviolet (UV) and visible spectral range (200–1600 nm) were acquired using optical integrating sphere spectrometer (Cary 5000, Agilent Technologies, Santa Clara, CA, USA). Raman measurements were undertaken with 532-nm wavelength CW laser fiber-coupled to micro-Raman microscope (Alpha 300, WiTec GmbH, Ulm, Germany). Each representative single spectrum was averaged over 50 times at different sample sites, while the signal integration time was 180 s. In the mapping regime, the signal integration time in each spot was 1 s. Pump laser intensity was chosen to avoid any possible laser-induced modification of α-Si even after several minutes of exposure.

## 3. Results and Discussions

Laser irradiation of sample surfaces was performed at fixed pulse energy *E*${}_{p}$ of 1.5 μJ and sample scanning speed *V* of 1 mm/s. At maximal available pulse repetition rate, such processing parameters ensured gentle laser annealing (crystallization) of the material without formation of so-called laser-induced periodic surface structures induced by corresponding in-plane modulation of the laser intensity [43,44,45]. Such a modulation oriented along the polarization vector was justified to originate from interference of the incident and scattered laser radiation. The surface morphology modification (typically oxidation, [42]) is known to start at interference maxima where laser intensity reaches the corresponding threshold fluence. It should be noted that faster scanning speed at such a fluence provided no visible laser-induced modification, indicating threshold behavior of the process. Noteworthy, the minimal temporal distance between two neighboring pulses defined by the pulse repetition rate used in our experiments was several orders of magnitude longer compared with the duration of characteristic laser-induced processes occurring in amorphous films, such as thermalization/melting (electron-phonon coupling) and resolidification which are within picosecond and nanosecond timescale [46], respectively. In this respect, the other laser processing parameters (pulse energy and scanning speed) can be easily optimized for smaller repetition rates to achieve similar annealing of α-Si films.

Furthermore, considering processing parameters used (*F*${}_{l}$ = 150 mJ/cm${}^{2}$, λ = 1026 nm, τ = 230 fs, *f* = 200 kHz), the maximal temperature on the film surface was estimated to be about 700 ${}^{\circ }$C according to ref. [47]. This value is significantly lower than the melting point of silicon (≈1400 ${}^{\circ }$C) and corresponds to the temperature associated with solid-phase crystallization processes [48]. Importantly, owing to low thermal diffusion under fs-laser excitation, the actual temperature near the film-substrate interface can be even lower [4], which makes the present technique promising for LTPS fabrication on polymer substrates. Laser-annealed α-Si film areas processed at optimal parameters demonstrate changed optical properties, which can be seen in optical reflection and transmission images (Figure 1b). In particular, the yellowish color in the transmission optical image indicates corresponding modification of the transmission properties of the laser-treated area with respect to those of pristine sample. Optical photography (Figure 1c) of the glass-supported α-Si film also illustrates higher transmission within a rather large (≈1 cm${}^{2}$) rectangular-shaped laser-processed area that was produced at optimal parameters by merging line scans at a step of 30 μm.

**Figure 2:**
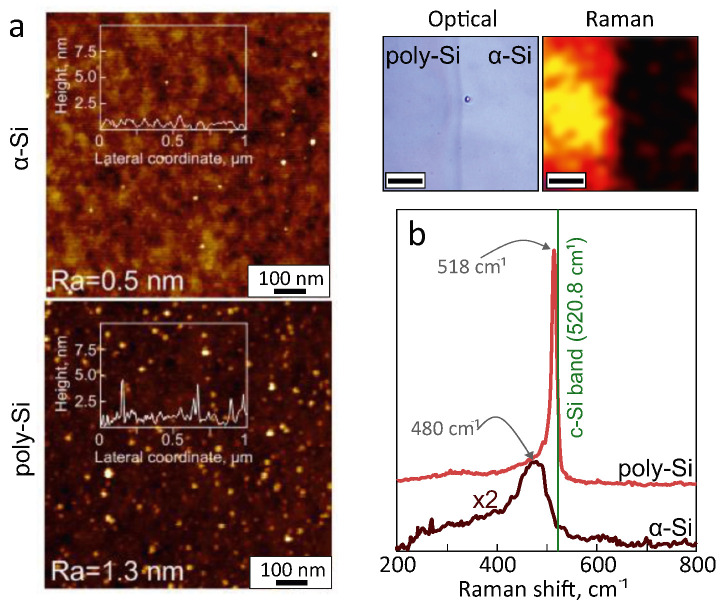
(**a**) AFM images revealing surface morphology and roughness of pristine α-Si and laser-annealed poly-Si films. (**b**) Raman spectra of pristine α-Si and laser-annealed poly-Si. Insets in (**b**) provide correlated contrasted optical and micro-Raman (@518 ± 3 cm${}^{-1}$) images taken near the boundary between pristine and annealed poly-Si. Scale bar in both images indicates 7 μm.

Importantly, SEM inspection of the poly- and α-Si surface indicated no significant changes of surface morphology that could be observed. Comparative AFM analysis revealed a very small increase in surface roughness (R${}_{a}$) from 0.5 to 1.3 nm upon laser processing of pristine α-Si film (Figure 2a). Such roughening can be partially attributed to a certain amount of nanoparticles that can be generated and deposited from the surface areas processed during dose tests. Still, this level of surface roughness is at least one order of magnitude lower than what was reported previously for α-Si films annealed by near-IR fs-laser pulses at room temperature in air (R${}_{a}$∼28 nm [34] or ∼150 nm [40]). It was demonstrated earlier that surface roughness increases with laser fluence [41], however, the fluence of 150 mJ/cm${}^{2}$ used in this work is higher than that used in work [34], where it was about 70 mJ/cm${}^{2}$. So, the reasons for significantly lower R${}_{a}$ may be the smoother initial α-Si surface and lower scanning speed. To evaluate chemical composition, EDX analysis was performed at an accelerating voltage of 3 kV to minimize contribution from the substrate. The results revealed a negligible increase of the oxygen fraction in the laser-annealed area (from 6.5 to 7.5 at.%), potentially indicating formation of a nanometer-thick oxidized layer (the atomic percentage of Si was detected as 93%).

Raman microspectroscopy was used to further probe crystallinity of the laser-annealed areas. Representative averaged Raman spectra of pristine α-Si and poly-Si are shown in Figure 2c. If compared with untreated α-Si film that only demonstrated a low-intensity broad Raman band centered at 480 cm${}^{-1}$, the laser-annealed poly-Si shows a sharp peak at 518 cm${}^{-1}$ which is slightly shifted with respect to the reference Raman frequency of monocrystalline Si at 520.8 cm${}^{-1}$ (shown by a vertical line in Figure 2c). This spectral shift is indicative of nanocrystalline Si phase with the estimated size of nanograins around ≈10 nm according to ref. [49]. Mapping the intensity of this Raman band (518 ± 4 cm${}^{-1}$) near the boundary between pristine and laser-annealed areas allowed us easily to separate poly-Si and α-Si phase areas (see top insets in Figure 2c).

Further, to reveal the difference in optical constants (refractive index, *n*, and absorption coefficient, *k*) between the pristine and laser-annealed films, we measured their UV-vis-IR reflection/transmission spectra. For both samples, their transmission/reflection coefficients were found to demonstrate typical Fabry-Perot modulations. However, for the laser-annealed poly-Si the modulation amplitude increases (especially in the visible spectral range), indicating a certain decrease of *k*. Moreover, a distinct blueshift of the reflection/transmission spectra observed for the poly-Si film (with respect to those of the pristine α-Si) indicates corresponding change of the *n* value.

**Figure 3:**
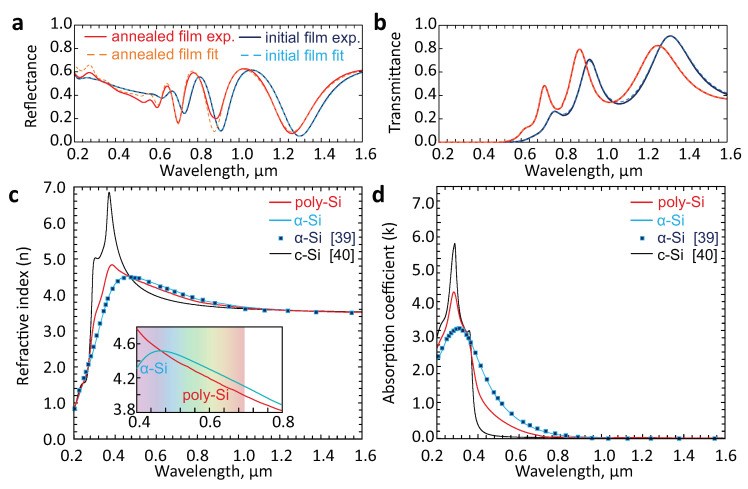
(**a**,**b**) Measured and calculated reflection and transmission spectra of pristine α-Si and laser-annealed poly-Si films. Solid curves provide experimental data while dashed curves present numerical fit. (**c**,**d**) Refractive index (*n*) and absorption coefficient (*k*) of pristine α-Si (blue solid curve) and laser-annealed (red solid curve) poly-Si film calculated from experimentally measured optical spectra. Inset in (c) highlights difference in refractive index of pristine and laser-annealed films in the visible spectral range. Markers in (c,d) give reference data for amorphous Si (α-Si, [52] ) and monocrystalline Si (c-Si, black curve, [53] ).

Based on the obtained reflection/transmission spectra, both the refractive index *n* and absorption coefficient *k* were further evaluated using common photometric approach [50,51] (see also Methods for details). The results of these calculations are summarized in Figure 3c,d along with the reference data for amorphous [52] and monocrystalline bulk silicon [53]. Noteworthy, the obtained dispersion curves for pristine α-Si film perfectly fit the corresponding reference data [52], indicating correctness of the measurements and calculations. Also, transmission spectra obtained with such a numerical fit demonstrate excellent agreement with the experimental data (see dashed curves in Figure 3a). As seen in Figure 3, for the laser-annealed poly-Si film the absorption coefficient decreases twice in the visible spectral range. Its obtained refractive index also decreases with respect to that of the pristine film by about 0.1 refractive index units (RIU) in the visible spectral range (at wavelength >500 nm) and increases largely in the blue part of the spectrum, following the trend similar to dispersion curve for monocrystalline Si. These features are indicative for polycrystalline silicon and are generally consistent with the previously reported studies for laser-annealed films [54]. Systematic measurements of different sites on the surface of the poly-Si sample also indicated the average thickness of the annealed film to be of 360 ± 5 nm, which is close to that of the pristine α-Si. A small decrease of the height may imply film shrinkage upon its annealing [55]. However, a similar height deviation was found by probing optical spectra for the pristine α-Si. Also, AFM measurements carried out near the boundary between poly- and α-Si (shown as inset in Figure 2c) reveal no detectable height modulations [56]. In this respect, the shrinkage cannot be reliably identified in our experiments.

Finally, the gentle fs-laser annealing applied is seen to cause a minimal effect on the morphology of silicon films as confirmed by SEM/AFM measurements. In combination with a significant changes of the refractive index (≈0.1 RIU; see inset in Figure 3c) and a considerable increase of transmission coefficient (decrease of *k*) in the yellow-red spectral range, this feature makes direct fs-laser processing of inexpensive α-Si films promising for optical information storage, anti-counterfeiting and recording of various planar micro-optical elements (for example, waveguides, Fresnel lenses and diffraction gratings). However, at this point, demonstration of such elements and their performance is out of scope of this paper and will be a subject of our forthcoming studies.

**Figure 4:**
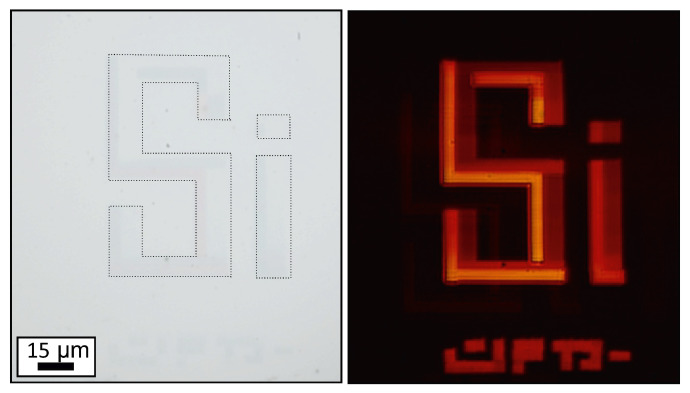
Reflection (**left**) and transmission (**right**) optical images of α-Si film containing laser-annealed microscale poly-Si areas arranged to form “Si” letters. Black dashed lines highlight the location of laser-annealed area in the reflection image.

Nevertheless, in the end of this report, we demonstrate how fs-laser radiation can drive local crystallization of α-Si at the microscale, opening pathways for the above mentioned applications. We took advantage of a considerable decrease of *k* for poly-Si to encrypt “Si” letters in the pristine film via localized laser annealing (Figure 4). These letters can hardly be resolved in the reflection-mode optical image appearing only under their visualization in transmission regime.

## 4. Conclusions

In conclusion, this work demonstrates that near-IR femtosecond laser pulses can efficiently drive phase transition in glass-supported 365-nm thick α-Si, resulting in formation of high-quality uniform polycrystalline Si. Composition and crystallinity of the obtained poly-Si film was verified with Raman and energy-dispersive X-ray spectroscopy, while comparative atomic-force microscopy analysis of the as-deposited α-Si and produced poly-Si films revealed very small increase of surface roughness from 0.5 to 1.3 nm upon laser texturing. Optical spectroscopy supported by numerical modeling confirmed improved optical characteristics of the laser-annealed poly-Si films in the visible and near-IR spectral range, which is associated with lower losses in the poly-Si phase. Direct femtosecond processing can be optimized to uniformly anneal α-Si over large-scale surface area or drive phase transition in a local volume. In particular, tight focusing of fs-laser radiation with high-NA optics is expected to provide a lateral resolution that is close to radiation wavelength (1030 nm in our experiments), thus opening pathways for fabrication of IR micro-optics and optical information encryption at 25,000 dpi. Chemical etching combined with laser processing can further enrich potential designs. Moreover, laser beam shaping with diffraction optical elements or spatial light modulators will apparently permit to optimize laser annealing process via proper laser energy delivery into α-Si material [57].
